# Hæmoglobinuria

**Published:** 1894-02

**Authors:** 


### HÆMOGLOBINURIA.

### Extract from Letter of John M. Eshleman, V.M.D.

Hæmoglobinuria (azoturia) is not so frequent here as in cities, but I get over 90 per cent. well by the use of tr. opii and chloral hyd. every 4 hours ; F. E. buchu and jaborandi in full doses every 3 hours; and aloes in full quantity. Whether it is due to the treatment or not, I will not say, but they get well. The last two cases I had to die I withdrew opium and chloral, when pain ceased; in two days they took worse and died.

